# Antidiabetic Effects of Resveratrol: The Way Forward in Its Clinical Utility

**DOI:** 10.1155/2016/9737483

**Published:** 2016-12-05

**Authors:** Omolola R. Oyenihi, Ayodeji B. Oyenihi, Anne A. Adeyanju, Oluwafemi O. Oguntibeju

**Affiliations:** ^1^Department of Biochemistry, Bowen University, Iwo, Nigeria; ^2^Discipline of Biochemistry, School of Life Sciences, University of KwaZulu-Natal, Westville Campus, Private Bag X54001, University Road, Durban 4000, South Africa; ^3^Nutrition and Chronic Disease Research Unit, Oxidative Stress Research Centre, Department of Biomedical Sciences, Faculty of Health and Wellness Sciences, Cape Peninsula University of Technology, Bellville, South Africa

## Abstract

Despite recent advances in the understanding and management of* diabetes mellitus*, the prevalence of the disease is increasing unabatedly with resulting disabling and life-reducing consequences to the global human population. The limitations and side effects associated with current antidiabetic therapies have necessitated the search for novel therapeutic agents. Due to the multipathogenicity of* diabetes mellitus,* plant-derived compounds with proven multiple pharmacological actions have been postulated to “hold the key” in the search for an affordable, efficacious, and safer therapeutic agent in the treatment of the disease and associated complications. Resveratrol, a phytoalexin present in few plant species, has demonstrated beneficial antidiabetic effects in animals and humans through diverse mechanisms and multiple molecular targets. However, despite the enthusiasm and widespread successes achieved with the use of resveratrol in animal models of* diabetes mellitus*, there are extremely limited clinical data to confirm the antidiabetic qualities of resveratrol. This review presents an update on the mechanisms of action and protection of resveratrol in* diabetes mellitus*, highlights challenges in its clinical utility, and suggests the way forward in translating the promising preclinical data to a possible antidiabetic drug in the near future.

## 1. Introduction

Resveratrol (3, 5, 4′-trihydroxy-*trans-*stilbene) is a nonflavonoid polyphenolic compound belonging to the stilbenoid class. It is the most relevant among stilbenes, due to its well-known bioactivity. It was first identified in 1940 by Michio Takaoka from hellebore roots (Veratrum grandiflorum O. Loes) and it was from this source that the name “resveratrol” was derived [1]. In 1963, Nomomura also isolated it from the Japanese knotweed* Polygonum cuspidatum* before Siemann and Creasy later discovered it in wine in 1992. Resveratrol (RSV) is a natural phytoalexin found in wine. The consumption of red wine was linked to the low mortality of the French population and the term “French Paradox” was used to explain the observation of low mortality despite high risk of cardiovascular diseases (CVD). However, RSV is present at very low concentration [2], suggesting that the tag on RSV, “the red wine molecule,” was overhyped. Regardless of whether or not the French Paradox involves RSV, a plethora of preclinical and clinical studies have demonstrated its beneficial effects [3]. RSV can also be found in grapes, variety of berries, peanuts, soy, and dark chocolate at different concentrations as shown in Table 1.

**Figure 1:**
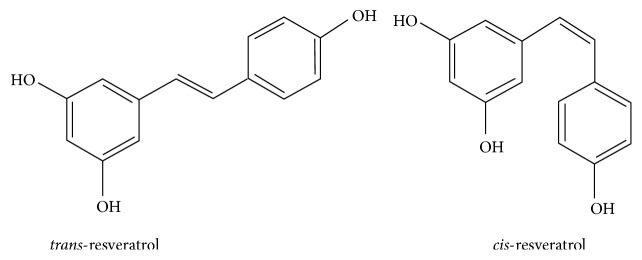
Chemical structures of resveratrol.

Structure-activity relationship seems to be crucial in the determination of the biological activities of RSV. The chemical structure of RSV consists of two aromatic rings linked by a methylene bridge and has been reported to exist in* cis*- and* trans*-stereoisomeric forms (Figure 1). However, the* trans*-isoform is the biologically active type [4]. During its metabolism, RSV undergoes a biotransformation by CYP1B1, a cytochrome P450 enzyme, to its monohydroxylated form, piceatannol [5]. This metabolite was found to possess a stronger activity than RSV probably due to the presence of more hydroxyl groups that led to increase in inhibitory activities on nuclear factor kappa-B (NF-*κ*B) [6] and its inducer activity on apoptosis [7]. Ovesná and Horváthová-Kozics [8] described that trans-stilbene compounds containing ortho-diphenoxyl or para-diphenoxyl functional groups with 4′-hydroxyl group and double bond presented a high chemoprevention. In addition, the number and position of carboxyl groups, intramolecular hydrogen bonding, stereoisomerism, and presence of double bonds have been reported to influence its biological activity.

RSV has elicited beneficial effects in cardiovascular disorders, cancer, diabetes, obesity, amyloidogenesis, and others [9–13]. Some of these beneficial effects occurred* via* mechanisms such as anti-inflammation [14]; antioxidation [15]; antihyperglycaemia [16]; vasodilation [17]; and clearance of amyloid *β* (A*β*) peptides [18]. The molecular targets of RSV may include sirtuin 1 (SIRT1), adenosine monophosphate activated kinase (AMPK) [19–23], nuclear factor-kappa B (NF-*κ*B) [22, 24, 25], and kelch-like ECH-associated protein 1 (Keap-1)/nuclear factor erythroid 2-related factor 2 (Nrf2) [24].

**Figure 2:**
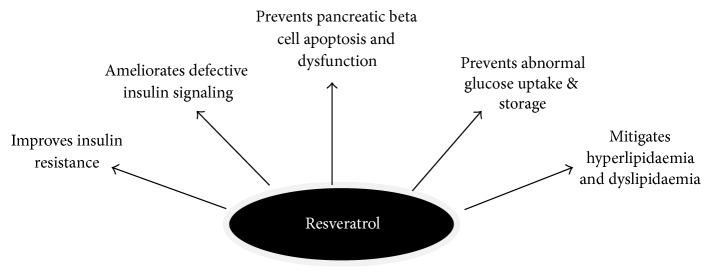
Protective effects of resveratrol in* diabetes mellitus*, adapted from [59, 64, 71, 120–122].

The high safety profile of RSV, coupled with its multidirectional effects, has stimulated the interest of the scientific community making it an attractive candidate in the protection against the deleterious effects of hyperglycaemia. A large body of evidence has shown that RSV exerts protective effects in DM* via* several mechanisms as summarized in Figure 2. Despite the numerous preclinical data on the antidiabetic effects of RSV, there is limited evidence on its beneficial effect in diabetic humans. The aim of this review is to present an update on the evidence supporting the antidiabetic effects of RSV in DM and directions for future research.

## 2. *Diabetes Mellitus*

*Diabetes mellitus* (DM), a long-term, multifactorial, metabolic disease of severe complications, remains a global health challenge in the 21st century. According to a recent estimate of the International Diabetes Federation (IDF), DM has caused approximately 5 million deaths in the year 2015 out of the 415 million adults suffering from the disease [26]. The increasing morbidity and mortality rates observed among diabetic patients are majorly due to the high incidence and severity of diabetic complications which occur due to lack of proper management and persistent hyperglycaemia in DM. Uncontrolled progression of DM leads to the developing damages in the liver, kidney, brain, retina, heart, nerve, and blood vessel [27]. The current therapeutic options employed in the management of the disease have serious limitations due to adverse effects and reduced efficacy over time. Therefore, the search for more efficient antidiabetic drugs is on-going.

DM arises from major defects in insulin secretion, activities, or both [28]. The two major types are insulin-dependent* diabetes mellitus* (IDDM-type 1) and non-insulin-dependent* diabetes mellitus* (NIDDM-type 2). The former is characterized by selective autoimmune destruction of insulin producing pancreatic *β*-cell and insulin deficiency whereas the latter is a case of insulin resistance coupled with impaired insulin secretion [29]. It is evident that the inability of the pancreatic *β*-cells to produce enough effective insulin to maintain glycaemic control is a feature of both types of DM. Autoimmunity, genetic makeup, and environmental factors are responsible for pancreatic islets destruction [30] whereas obesity, nutritional disorders, hypertension, lifestyle habits, and genetic/hereditary factors have been implicated in insulin resistance and type 2 DM through their effects on glucose metabolism [14].

Hyperglycaemia (a major characteristic of type 1 DM) results when the rate of glucose production far exceeds its utilization and storage. The result is a persistent increase in blood glucose level [31]. The high blood glucose pool is a consequence of some underlying predisposing factors such as inadequate insulin secretion by autoimmune-induced partially destroyed pancreatic *β*-cells, ineffective sensitization, and activation of insulin receptors/signal messengers. Overall, these factors consequently lead to derangements in metabolic processes such as glucose, lipid, and protein metabolism. Factors that aggravate the hyperglycaemic state involve increased mobilization of liver glycogen stores and hepatic gluconeogenesis, impaired glucose utilization, rapid mobilization of triglycerides, dysregulation of protein catabolism, and elevated blood levels of glucogenic amino acids which serve as gluconeogenic precursors [32].

Although the precise aetiopathogenesis of DM is still under debate, oxidative stress, inflammatory factors, and autoimmune reactions have all strongly emerged as the major pathogenic effectors for DM [33]. Oxidative stress results from overproduction of reactive oxygen species (ROS) and reactive nitrogen species (RNS) or the inadequate disposal of these species by antioxidants resulting in multiple deleterious effects on cellular metabolism. An antioxidant has been defined as any substance that when present at low concentration compared with those of an oxidizable substrate significantly delays or prevents oxidation of that substrate [34]. When an imbalance ensues between oxidant generation and antioxidant defence, the consequence is oxidative stress. Enormous evidence has emerged to support the role of oxidative stress in the pathogenesis of DM and in the development of vascular complications [31, 35]. Excessive elevation of blood glucose directly causes *β*-cell stress, impairment of *β*-cell function, and survival, through generation of ROS and RNS [36]. Generation of these free radicals in DM can occur through mitochondrial superoxide overproduction, nonenzymatic glycation of proteins, glucose oxidation, overactivity of the hexosamine pathway, and activation of protein kinase C isoforms [37]. Excess ROS has the capacity to attack DNA, lipids, proteins, and carbohydrates forming harmful products/complexes such as advanced lipid and glycation end-products that cause deleterious consequences in the body [38]. The generation of excess free radicals, lipid peroxidation products, protein/carbohydrate oxidation products, and DNA adducts has been observed in a variety of* in vitro* and* in vivo* diabetic animal models [39].

Free radicals can also be produced by inflammatory cells at the site of inflammation leading to exaggerated oxidative stress [40]. Free radicals can initiate intracellular signaling cascade that enhances the synthesis of inflammatory mediators. Inflammation and oxidative stress are therefore “essential partners” in DM as both processes contain mechanisms for mutual amplification. Therefore, it has been proposed that a candidate antidiabetic drug should be one that possesses polypharmacological abilities due to the multipathogenicity of DM.

## 3. Mechanisms of Protection of Resveratrol in* Diabetes Mellitus* (Preclinical Data)

**Figure 3:**
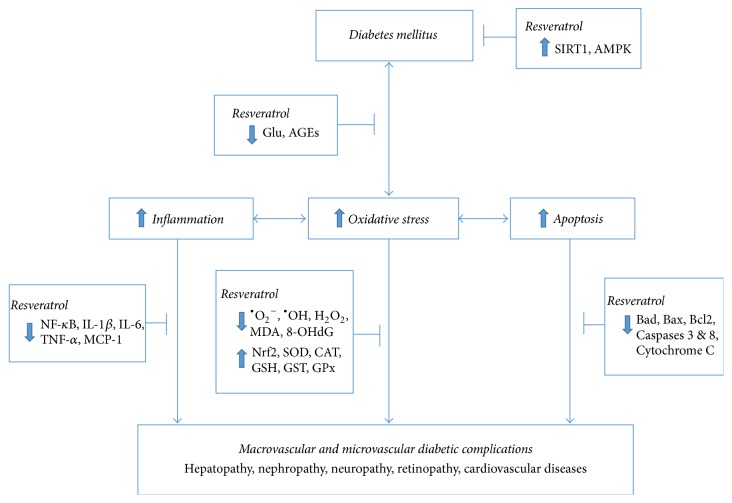
A simplified scheme showing mechanisms of resveratrol protection against the development of diabetic complications. SIRT1, sirtuin-1; AMPK, adenosine monophosphate activated kinase; Glu, glucose; AGEs, advanced glucose end-products; ^∙^O_2_^−^, superoxide anion; ^∙^OH, Hydroxide radical; H_2_O_2_, hydrogen peroxide; MDA, malondialdehyde; 8-OHdG, 8-hydroxydeoxyguanine; Nrf2, nuclear factor erythroid 2-related factor 2; SOD, superoxide dismutase; CAT, catalase; GSH, reduced glutathione; GST, glutathione S-transferase; GPx, glutathione peroxidase; NF-*κ*B, nuclear factor-kappa B; IL, interleukin; TNF, tumour necrosis factor; MCP, monocyte chemoattractant protein; Bcl2, B-cell lymphoma 2; Bad, Bcl-2-associated death promoter; Bax, bcl-2-like protein 4.

RSV has been reported to mitigate the harmful effects of DM in several* in vitro* and* in vivo* animal experiments through divergent mechanisms [32]. These mechanisms range from the reduction of blood glucose concentration (by increasing glucose uptake, utilization, and storage and increasing insulin sensitivity) and the restoration of abnormal insulin signaling pathways (by silencing the transcription of some genes or inactivation of proteins). The development of complications such as nephropathy, neuropathy, retinopathy, and cardiovascular diseases is primarily responsible for the increase in morbidity and mortality rates among diabetic individuals worldwide [27, 41]. Several interconnected pathways have been postulated to hasten the progression of DM to diabetic complications. Some protective mechanisms through which RSV act against these pathways are illustrated in Figure 3. Studies have shown that RSV improves biochemical and clinical parameters in both type 1 and type 2 animal models of diabetic nephropathy [25, 42, 43]; diabetic neuropathy [3, 44, 45]; diabetic retinopathy [20, 46–48]; diabetes-induced hypertension [49, 50]; diabetes-induced cardiovascular diseases [22, 51, 52]; and diabetes-induced liver injury [53, 54].

### 3.1. Mechanisms to Combat Hyperglycaemia

High blood glucose concentration has the capacity to destroy pancreatic *β*-cells resulting in significant reduction of insulin secretion. As a mechanism to reduce hyperglycaemia, RSV has been shown to promote an increase in pancreatic *β* cell population [55] and trigger an increase in the secretion of insulin [56, 57]. As more insulin is secreted, excess blood glucose may be either stored as glycogen or utilized by tissues until glucose homeostasis is restored and maintained. The induction of glycogenesis in liver and skeletal muscle has been reported in diabetic rats following RSV administration [58]. Also, RSV treatment causes an increase in glucose uptake* via* the translocation of GLUT-4 to the membrane in diabetic rats [59]. Furthermore, RSV has been demonstrated to ameliorate hyperglycaemia-mediated disturbances in lipid and protein metabolism in rats supporting its antidiabetic actions [54, 60].

Impaired glucose tolerance (IGT) is a condition where the elevation of blood glucose level is not considered high enough to be classified as DM. This condition normally precedes a full-blown hyperglycaemia. It has been postulated that an intensive management of IGT in susceptible individuals may be critical in preventing the onset of DM [61]. In high-fat diet-fed mice, RSV was shown to significantly improve glucose tolerance [62, 63]. Similar improvement in glucose tolerance has been reported in diabetic rats [64, 65] and this effect has been proposed to occur primarily through AMPK-dependent mechanisms [66]. The ability of RSV to improve oral glucose tolerance has also been demonstrated in normal nonhuman primates chronically fed with RSV-supplemented diet [67].

Dysregulated insulin signaling is an integral factor in the development of insulin resistance and type 2 DM [68]. Excess blood glucose level increases the production of free radicals and advanced glycated end-products (AGE) that may inhibit proteins and enzymes involved in the insulin signaling pathways leading to the development of insulin resistance. Also, AGE has been shown to trigger stress signals that modify insulin receptor substrate proteins by increasing their serine/threonine phosphorylation and are subsequently destroyed partly contributing to insulin resistance [69]. RSV has been reported to induce p-Akt expression in STZ-diabetic rats [59, 70]. Furthermore, RSV was recently shown to improve IRS-1 mediated insulin signaling through inhibition of protein tyrosine phosphatase (PTP) 1B expression in liver and muscle of diabetic rats [71]. In addition, RSV was reported to downregulate the expression of receptor for AGE (RAGE) in diabetic rat tissues [72, 73]. Therefore, RSV amelioration of AGE-induced dysregulated insulin signaling through the inhibition of AGE production and activities may partly contribute to its antihyperglycaemic property in DM.

RSV may exert its antihyperglycaemic effects through the activation of SIRT1. SIRT1, an NAD^+^-dependent deacetylase, has been described as an important regulator of many factors influencing type 2 DM [21, 63, 74]. Studies have revealed that SIRT1 activity and expression were decreased significantly in* in vitro* and* in vivo* experimental models of DM [75, 76]. However, the administration of RSV was reported to normalize hyperglycaemia in obese mice with DM partly through the activation of SIRT1 [77]. In a recent study, an intraduodenal infusion of RSV improved insulin sensitivity and lowered hepatic glucose production in three rat models of insulin resistance [78]. The glucoregulatory effects of RSV in the study were attributed to the activation of SIRT1 and AMPK. The results from the same study were further supported by a development of hepatic insulin resistance in rats fed normal chow following a duodenum-specific knockdown of SIRT1 expression for 14 days.

The antidiabetic effects of RSV may be linked to the activation of SIRT1 mechanistic pathways. Upon activation, SIRT1 was reported to deacetylate forkhead box protein (FOX) O1, inhibiting its activity and consequently suppressing pancreatic *β*-cell apoptosis [79]. The FOXO1-mediated induction of transcription factors MafA and NeuroD, which play important roles in the survival of pancreatic *β*-cells, may be sustained by the activation of SIRT1. This action results in enhanced insulin sensitivity and improved regulation of glucose homeostasis [80]. In addition, SIRT1 has been shown to deacetylate PTP1B reducing its activity and leading to the improvement of insulin sensitivity in insulin-resistant conditions [81]. In experimental animals, activation of SIRT1 was reported to offer protection against obesity and insulin resistance by positively regulating the secretion of insulin [82]. The regulation of insulin secretion by SIRT1 has also been shown to trigger glucose uptake and utilization [83]. Experimental studies in pancreatic *β*-cells in mice have revealed an improvement in glucose tolerance and enhancement of insulin secretion when the dose of SIRT1 was increased [84, 85]. The stimulation of insulin secretion by SIRT1 has been suggested to occur* via* transcriptional repression of uncoupling protein 2 (UCP2). In pancreatic *β*-cells, the overexpression of SIRT1 has been shown to increase ATP* via* the repression of UCP2 leading to cell membrane depolarization and Ca^2+^-dependent insulin exocytosis [86].

Furthermore, some of the beneficial effects of RSV on the regulation of glucose homeostasis may be mediated through the activation of AMPK. AMPK regulate several important intracellular processes such as energy metabolism, mitochondrial functions, and cellular homeostasis [32]. AMPK is activated mainly by a low cellular energy status (high AMP/ATP ratio), elevated intracellular nucleotide adenine dinucleotide (NAD^+^) concentration, and the catalytic actions of cyclic nucleotide phosphodiesterase (PDE). Energy stress conditions like diet restriction, hypoxia, exercise, and fasting are very strong factors for the activation of AMPK [87]. Once activated majorly by phosphorylation, AMPK promotes the inhibition of processes that utilize ATP or inhibits ATP production in the cell. Under hyperglycaemic conditions, the dysregulation of AMPK activity correlated with insulin resistance and hyperglycaemia-associated tissue damage [88], supporting a key role of AMPK in type 2 DM. The effects mediated by RSV* via* AMPK activation include the regulation of insulin sensitivity, increase in glucose uptake, and regulation of insulin secretion in pancreatic *β*-cells [66]. The alleviation of diabetic complications upon the activation of AMPK by RSV has been confirmed in animal models [15, 19, 59, 89]. Also, AMPK activation has been shown to be initially triggered by RSV-induced activation of SIRT1 leading to increased mitochondrial biogenesis and function [90].

### 3.2. Mechanisms to Combat Oxidative Stress

One of the main mechanisms responsible for the antidiabetic effects of RSV involves its antioxidant actions. In diabetic rat tissues, RSV has been reported to normalize the concentration of oxidative stress indicators such as superoxide anion (O_2_^∙−^), hydroxyl radical (OH^∙^), hydrogen peroxide (H_2_O_2_), malondialdehyde (MDA), thiobarbituric acid reactive substances (TBARS), 8-isoprostane, 8-hydroxydeoxyguanine (8-OHdG), nitro-tyrosine (nitro-Tyr), reduced/oxidized glutathione (GSH/GSSG) ratio, and nitrite/nitrate ratio [24, 52–54, 91–93]. Furthermore, RSV has been reported to exhibit its antioxidant activities by increasing the activities of antioxidant enzymes such as superoxide dismutase (SOD), catalase (CAT), glutathione peroxidase (GPx), glutathione reductase (GRed), glutathione S-transferase (GST), and quinone reductase (QRed) as well as increasing the levels of nonenzymatic antioxidant compounds (reduced glutathione and vitamins C and E) in diabetic animals [24, 48, 52, 53, 94].

Apoptotic cell death is a consequence of hyperglycaemia-mediated oxidative stress. The effects of RSV on apoptosis in the kidney [95], heart [52], brain [96], testes [97], retina [48], and muscle [98] in diabetic animals have been reported. In these studies, RSV was shown to significantly reduce the apoptotic index coupled with a reduction in the levels of proapoptotic proteins in diabetic tissues.

In addition to the central role of SIRT1 in the regulation of glucose metabolism, mitochondrial function, and respiration biogenesis, it is also involved in cellular processes such as apoptosis, inflammation, oxidative stress, calorie restriction, and aging [3, 87, 99, 100]. In type 1 DM, oxidative stress resulting from an increased generation of O_2_^∙−^ accompanies the reduction in SIRT1 expression. Hence, activation of SIRT1 is a key protective effect against oxidative stress in type 1 DM. There have been reports indicating that the activation of SIRT1 by RSV suppresses ROS generation* via* the enhancement of the activity of FOXO3a [101] and upregulation of MnSOD expression [102].

Another molecular target of RSV is Nrf2 (nuclear factor erythroid 2- [NF-E2-] related factor 2), a transcription factor responsible for the constitutive and inducible expression of antioxidant response element- (ARE-) regulated genes [103]. The activation of the Nrf2/ARE pathway plays a major role in the protective action exerted by RSV against oxidative stress in DM by upregulation of cellular antioxidant defence mechanisms including heme oxygenase-1 (HO-1) and GPx [102]. RSV treatment of diabetic rats normalized the decreased renal expression of Nrf2 in hyperglycaemia-mediated oxidative stress and upregulated its downstream regulatory proteins such as gamma-glutamylcysteine synthetase (*γ*-GCS), *μ*-GST, and HO-1 [24]. Under nonactivated conditions, Nrf2 interacts with Keap-1, thereby limiting Nrf2-mediated gene expression. Upon activation by RSV, the Keap-1-Nrf2 complex dissociates and Nrf2, which is released from Keap-1, binds to ARE to upregulate the expression of genes in order to reduce oxidative stress.

### 3.3. Mechanisms to Combat Inflammation

As part of a coordinated response to the onslaught of ROS and other cellular stress signals, inflammatory cells (such as monocytes/macrophages, lymphocytes, platelets, granulocytes, and mast) are recruited. These cells propagate their activities through the activation of inflammatory mediators such as cytokines/chemokines, cell adhesion molecules, growth factors, and prostaglandins [104]. Altogether, the overactivation of these processes acts act synergistically to accelerate the severity of the clinical manifestations of DM and diabetes-related complications.

RSV has been shown to elicit its anti-inflammatory properties in DM mainly* via* the inhibition of the nuclear factor NF-*κ*B pathway. NF-*κ*B is a proinflammatory master switch, which activates proinflammatory cytokines gene expression and apoptosis cascade [105]. Upon activation, NF-*κ*B can mediate the transcription of inflammatory genes. Production and release of proinflammatory cytokines and chemokines induce ROS and RNS production and further increase tissue oxidative stress resulting in a vicious cycle of inflammation and oxidative stress. Thus, modulation of NF-*κ*B may be an attractive treatment strategy in the treatment and management of DM. RSV administration has been reported to significantly decrease NF-*κ*B activity in the retinas of diabetic rats [48]. RSV also demonstrated inhibitory effect on NF-*κ*B activity and ameliorated the elevated levels of inflammatory proteins, TNF-*α* (tumour necrosis factor-*α*), interleukin- (IL-) 6, and COX-2 (cyclooxygenase 2), thus contributing to reduction in neuroinflammation and protection against functional and behavioural deficits in diabetic neuropathy [106]. The anti-inflammatory property of RSV has been confirmed in diabetic animals by the downregulation of proinflammatory genes and proteins like TNF-*α*, IL-1*β*, IL-6, IL-4, IL-17, COX-2 intercellular adhesion molecule 1 (ICAM), and vascular cell adhesion protein 1 (VCAM1) [14, 19, 22, 24, 107, 108] which are downstream targets of NF-*κ*B. In addition, RSV significantly decreased the activation and recruitment of macrophages thereby retarding the inflammation process in diabetic rats [22, 109]. Zhang and colleagues [110] reported that the anti-inflammatory effects of RSV in type 2 DM may be elicited* via* the downregulation of mRNA and protein expression of TNF-*α*, a known inflammatory trigger resulting in the inhibition of TNF-*α*-induced NAD(P)H oxidase activation, a key cellular source of O_2_^∙−^.

## 4. Clinical Studies on the Effects of Resveratrol in* Diabetes Mellitus*

Although numerous data exist on the beneficial outcomes of RSV in diabetic animals and* in vitro*, limited studies have specifically investigated the antidiabetic effects of RSV in humans [16, 111–113]. A vast majority of clinical studies on the effects of RSV have focused on healthy individuals or other metabolic disorders but there is paucity of comprehensive information on its effects in diabetic humans (Table 2). It is interesting to note that the data obtained from majority of the clinical studies carried out in DM have been consistent with the promising results from animal models. One of these studies also reported improvement in diabetes complication (foot ulcer) after RSV treatment [113].

Bhatt and colleagues demonstrated that RSV (250 mg/day for 3 months) administered along with glibenclamide and/or metformin demonstrated improvement in glycaemic parameters in diabetic patients [16]. The study reported improvement in HbA1c, systolic blood pressure, and total cholesterol in patients with type 2 DM treated with RSV combined with the oral hypoglycaemic agents. Brasnyó and colleagues [112] reported an improvement in insulin sensitivity in type 2 diabetic patients after treatment with a low dose of RSV (5 mg twice daily) for 4 weeks. In the study, RSV treatment was shown to decrease HbA1c, systolic blood pressure, and total cholesterol. A decrease in oxidative stress assessed by measuring urinary orthotyrosine excretion was also reported. However, the authors found no evidence that RSV influenced homeostasis model of assessment of *β*-cell function (HOMA-*β*) and therefore proposed that the mechanism of antidiabetic effects might be due to reduction in oxidative stress and a more efficient insulin signaling. Recently, Movahed and others [111] reported that 1 g/day of RSV supplementation for 45 days notably reduced fasting blood glucose, HbA1c, insulin, and systolic blood pressure. However, compared to the study of Brasnyó and colleagues [112] who observed no effect on HOMA-*β* at the administered dose, data from the study of Movahed et al. revealed a decrease in HOMA-*β* as well as homeostasis model of assessment of insulin resistance (HOMA-IR). Another important observation from the two studies is that RSV not only complemented but also provided additional protection over standard antidiabetic medication.

Some beneficial outcomes have also been reported with RSV treatment in nondiabetic humans. In obese subjects, Timmers and colleagues [114] reported significant improvement in the metabolic profile and general health after RSV supplementation for 30 days thereby describing RSV as a calorie restriction mimetic. RSV showed beneficial effects on glucose homeostasis and insulin sensitivity; reduced intrahepatic lipid (IHL) content and expression of inflammatory genes; and improved mitochondrial efficiency. These effects may be associated with the activation of AMPK and increased SIRT1 and PGC-1*α* protein content in the muscle [114]. In a double blind, randomized, placebo-controlled clinical trial, the effects of one-month RSV supplementation (40 mg/kg body weight) was assessed on endothelial response and inflammatory capacity in healthy subjects. A lowered expression of genes associated with inflammation and initiation of atherosclerosis such as* VCAM, ICAM, IL-8, and IFN-γ* was observed following incubation of human coronary artery endothelial cells with plasma from participants [115].

Although several studies have reported the beneficial effects of RSV in clinical trials, some other studies have also shown no benefit. The daily administration of RSV at a dose of 150 mg for 30 days improved metabolic parameters and modulated the putative molecular targets of RSV in obese men with no endocrine disorder [114]. However, its administration at higher doses (1000, 1500, 2000, and 3000 mg) in obese men with nonapparent endocrine disorder [116] or a diagnosed disorder [117, 118] for the same or longer treatment periods did not reveal beneficial effects on metabolic parameters and its known molecular targets. Also, single doses (250 and 500 mg) of orally administered RSV were shown to increase cerebral blood flow but revealed no effect on cognitive function in healthy humans [17]. However, RSV administration at a dose of 200 mg/day for 26 weeks was reported to improve memory performance in addition to improving glucose metabolism [119]. Therefore, the dose and/or duration of RSV administration may influence outcomes in clinical trials and may differ between preventative or therapeutic clinical studies.

## 5. Challenges in the Clinical Utility of Resveratrol in* Diabetes Mellitus*

Numerous studies on the biological activity of RSV have been carried out* in vitro* and in animal models but there is so little human evidence. Unfortunately, studies conducted so far on the clinical utility of RSV in DM have been confronted with several limitations. The inherent cellular mechanisms underlying the antidiabetic effects of RSV deduced from preclinical data were not investigated properly in most of the studies. Single doses of RSV were employed in the studies, resulting in the lack of a proper understanding of dose-response relationships.

Another limitation in the RSV clinical trials is sample size, involving only small cohorts of diabetic patients and the short duration of the trials. Long-term intervention studies are required to prove the efficacy of RSV and these are usually very expensive. RSV is a natural substance that is commercially available to everyone at a relatively cheap cost. This may partly explain the low interest of the pharmaceutical industry in investing funds on clinical studies to perform large-scale trials that would clarify the efficacy of RSV in DM.

In addition to the aforementioned limitations, the low bioavailability of RSV in humans poses another challenge in its use in DM [133]. The low bioavailability of RSV results from rapid glucuronidation, sulfation, and clearance from the body. This challenge is compounded by the fact that many bioavailability studies do not quantify tissue distribution and plasma bioavailability may not fully represent the RSV pool [134]. Therefore, the dosage required for optimal bioavailability for sufficient tissue distribution needs to be clarified. Human clinical trials are being performed currently without a full understanding of the optimal dosage protocols. Molecular target and efficacy of RSV may differ depending on the dose. Treatment with lower doses of RSV may activate SIRT1, whereas higher doses activate AMPK in a SIRT1- independent manner. For example, in RSV treatment of C2C12 cells, a mouse skeletal muscle cell line at a dose of 25 *μ*M was reported to activate AMPK in a SIRT1-dependent manner, whereas a higher dose of 50 *μ*M activated AMPK in a SIRT1-independent manner [90].

## 6. The Way Forward

There is a strong correlation between RSV metabolism and its effectiveness. New approaches to increase the bioavailability of RSV can help to actualise its potentials as a therapeutic agent in DM and related complications. One of such approaches is the coadministration of RSV with compounds that act as substrate for enzymes involved in RSV metabolism. UDP-glucuronosyltransferase (UGT) and sulfotransferase (SULT) are key enzymes of RSV metabolism which catalyse glucuronidation and sulfation of RSV, respectively, enhancing its clearance from the body [134]. The combined effect of RSV and compounds that act as substrate for these enzymes may significantly increase the bioavailability of RSV.

Piperine is one of such compounds and has been shown to improve the effects and bioavailability of RSV in cell cultures and animal models. However, the outcome of the clinical trial on coadministration of RSV and piperine so far failed to improve bioavailability [135]. The lower dosage of piperine used in the human study which prevented a saturation of the UGT enzyme and had no effect on the rate of metabolite formation has been suggested as the reason for the observed effect. The additive effect of piperine and RSV needs to be further investigated. In addition, there are reports on the synergistic or additive effects of RSV and other polyphenols such as quercetin, catechin, curcumin, and genistein, although implication on bioavailability has not been investigated in many of the studies [54, 136–139]. A more recent study demonstrated an increase in skeletal muscle mitochondrial capacity in healthy humans administered RSV (500 mg) and piperine (10 mg) supplementation as a bioenhancer to increase bioavailability and bioefficacy of RSV. However, a RSV only-supplemented group was not included in this study and bioavailability data was not available. Therefore, conclusions on improved effects and/or bioavailability of this combination in comparison to treatment with RSV only could not be reached [140].

In addition, the combination of RSV with genistein was reported to synergistically enhance antioxidant action in high-glucose treated Madin-Darby canine kidney (MDCK) epithelial cells when compared with RSV only and this combination may also have a therapeutic potential in diabetic nephropathy [141]. However, further studies are required to determine the effects of these combinations on the bioavailability of RSV and clinical relevance in diabetic conditions.

Long-term studies should be designed to determine appropriate dosage regimen and conclude on the antidiabetic effects of RSV in humans. Also, different new strategies such as the use of RSV nanoformulations, prodrugs, liposome-mediated delivery, and implantable devices could be used to improve the antidiabetic efficacy of RSV. Some of these technologies have been investigated in animal studies [142, 143] with no report in humans. RSV prodrugs can be developed for improved efficacy in diabetics. Metabolism of prodrugs into RSV in tissues of interest can maximize tissue concentration and can be beneficial in the treatment of tissue specific complications in diabetic patients. Targeted delivery of RSV prodrugs into tissues of interest* via* delivery systems such as liposome-mediated delivery or nanotechnological approaches may result in improved therapeutic outcome. Also, intravenous injection as an alternative to traditional oral route of administration of RSV may bypass gastrointestinal absorption, conjugation, and hepatic metabolism, thus resulting in increased bioavailability and improved outcomes in diabetic patients.

RSV analogues with improved bioavailability, which can be administered either alone or in combination with other antidiabetic drugs, should be developed. The RSV molecule could be the primer for the development of other synthetic compounds that can be more bioavailable and of interest to pharmaceutical companies. In the bioavailability studies of RSV, red blood cells and tissue samples (such as muscle and adipose biopsy) should be included when it is ethical and clinically possible for a better assessment of RSV pool and tissue distribution.

## 7. Conclusion

The divergent mechanism of actions of resveratrol* in vitro* and in animals coupled with the reported health benefits in type 2 diabetic humans should be a driving force for conducting clinical research. The time is ripe to embark on large, well-controlled clinical studies to confirm the efficacy of resveratrol in the management of* diabetes mellitus* and gain a better insight into its biological effects in humans. In addition, the effective doses of resveratrol and treatment duration may differ between preventative and therapeutic trials. Therefore, it is important to investigate the dose dependent effects at varying treatment periods. A combinational approach as well as improved formulations of resveratrol may help to overcome the challenge of maintaining an effective concentration at the site of action for an appropriate period. By considering the aforementioned factors limiting clinical utility of resveratrol in subsequent studies, we may be able to use resveratrol or its analogue to treat or prevent* diabetes mellitus* in humans in the nearest future.

## Figures and Tables

**Table 1:** Common sources of resveratrol and their estimated concentrations.

Sources of resveratrol	Resveratrol concentration
Bilberries	~16 ng/g
Blueberries	~32 ng/g
Cranberry juice	~0.2 mg/L
Cocoa powder	~1.85 *μ*g/g
Dark chocolates	~0.35 *μ*g/g
Milk chocolate	~0.10 *μ*g/g
White grape juice	~0.05 mg/L
Red grape juice	~0.50 mg/L
White grape seeds extract	0.25 mg/g
Red grape seeds extract	0.27 mg/g
White wines	<0.1–2.1 mg/L
Red wines	0.1–14.3 mg/L
Peanut	0.02–1.92 *μ*g/g
Peanut butter	0.3–1.4 *μ*g/g
Pistachios	0.09–1.67 *μ*g/g

Sources: [123–126].

**Table 2:** Clinical trials on the effects of resveratrol.

Subjects	Conditions of the subjects	Number of subjects	Dose and duration of study	Outcomes	References
Diabetic humans	Type 2 DM	62	250 mg/day + oral hypoglycaemic agents for 3 months	(i) Decreased HbA1c level (ii) No effects on body weight, high-density lipoprotein (HDL), and low-density lipoprotein (LDL) cholesterols	[16]
	Type 2 DM	70	500 mg twice daily for 45 days	(i) Decreased fasting blood glucose, HbA1c, insulin, and insulin resistance, (ii) Increased HDL level	[111]
	Type 2 DM	19	5 mg twice daily for 4 weeks	(i) Improved insulin sensitivity (ii) Increased pAkt : Akt ratio in the platelets	[112]
	Type 2 DM	24	50 mg twice daily for 60 days	(i) Reduced foot ulcer size and plasma fibrinogen level	[127]
	Type 2 DM and hypertensive patients with coronary artery disease	35	RSV-enriched grape extract for 12 months (8 mg/day in the first 6 month and 16 mg/day in the last 6 months)	(i) No effect on serum glucose level, HbA1c, lipids, and blood pressure (ii) Decreased IL-6 level and expression of IL-1*β* and TNF-*α*	[128]

Nondiabetic humans	Obese men with no family history of any endocrine disorder	11	150 mg/day oral administration for 30 days	(i) Reduced hepatic steatosis (ii) Decreased adipose tissue lipolysis (iii) Decreased intrahepatic lipid content, glucose, triglycerides, and inflammation markers (iv) Increased AMPK activation in muscle (vi) Increased SIRT1 level	[114]
	Obese men with no overt endocrine disorders	24	500 mg thrice for 4 weeks	(i) No detectable effects on insulin sensitivity, gene expression of inflammatory biomarkers, AMPK, and acetyl-CoA carboxylase	[116]
	Overweight/obese men with mild hypertriglyceridemia	8	2 weeks (1000 mg/day for 1st week followed by 2000 mg/day for 2nd week)	(i) No effect on insulin sensitivity, fasting, or fed plasma triglyceride concentration (ii) Reduced apoB-100 and apoB-48 production rates	[117]
	Overweight or obese men diagnosed with NAFLD	20	3000 mg/day daily for 8 weeks	(i) No effect on insulin resistance and steatosis (ii) No effect on plasma lipids or antioxidant activity. (iii) Increased levels of ALT and AST (iv) No effect on mRNA levels of *NQO1*, *PTP1B*, *IL-6*, or *HO-1*	[118]
	Nonobese, postmenopausal women with normal glucose tolerance	45	75 mg/day for 12 weeks	(i) No effect on insulin sensitivity in the liver, skeletal muscle, or adipose tissue (ii) No effect on plasma lipid or inflammatory markers (iii) No effect on the expression of *Ampk, Sirt1*, *Nampt*, and *Pgc-1α* in skeletal muscle or adipose tissue	[129]
	Healthy humans	22	250 and 500 mg for 2 study visits of few days apart	(i) Increased cerebral blood flow (ii) No effect on cognitive function	[17]
	Healthy humans	46	200 mg/day for 26 weeks	(i) Increased serum insulin level (ii) Decreased HbA1c (iii) Improved memory performance	[119]
	Healthy humans	44	400 mg RSV + 400 mg grape skin extract + 100 mg quercetin for 30 days	(i) Reduced plasma level of IFN-*γ* (ii) Decreased expression of ICAM, VCAM, and IL-8 in endothelial cells	[115]
	Elderly individuals with impaired glucose tolerance	10	Daily dose of 1, 1.5, or 2 g for 4 weeks	(i) Improved insulin sensitivity (ii) Reduced postprandial plasma glucose (iii) No effects on body weight, blood pressure, and lipid parameters	[130]
	Patients with stable angina pectoris.	166	20 mg/day for 60 days	(i) Decreased in serum levels of C-reactive protein (CRP) (ii) Decreased levels of lipid biomarkers	[131]
	Patients at high CVD risk on statins treatment for CVD prevention	75	12 months (8 mg/day in the first 6 months and 16 mg/day in the last 6 months)	(i) Decreased CRP, TNF-*α*, PAI type 1, and IL-6/IL -10 ratio (ii) Increased IL-10 level	[132]

ICAM: intercellular adhesion molecule; VCAM: vascular cell adhesion molecule; IL-8: interleukin-8; AMPK: adenosine monophosphate activated kinase; SIRT1: sirtuin-1; IFN-*γ*: interferon gamma; *NQO:* NAD(P)H:Quinone oxidoreductase; *Nampt*: nicotinamide phosphoribosyltransferase; *PGC*: peroxisome proliferator-activated receptor gamma coactivator; CRP: C-reactive protein.
